# Femicide and Attempted Femicide before and during the COVID-19 Pandemic in Chile

**DOI:** 10.3390/ijerph19138012

**Published:** 2022-06-30

**Authors:** Erika Cantor, Rodrigo Salas, Romina Torres

**Affiliations:** 1 Institute of Statistics, Universidad de Valparaíso, Valparaíso 2360102, Chile; 2 Millennium Institute for Intelligent Healthcare Engineering (iHealth), Santiago 7820436, Chile; rodrigo.salas@uv.cl; 3 Centro de Investigación y Desarrollo en Ingeniería en Salud, CINGS-UV, Universidad de Valparaíso, Valparaíso 2362905, Chile; 4 School of Biomedical Engineering, Universidad de Valparaíso, Valparaíso 2362905, Chile; 5 Faculty of Engineering, Universidad Andres Bello, Viña del Mar 2520584, Chile

**Keywords:** femicide, violence against women, COVID-19, risk factors

## Abstract

Experts and international organizations hypothesize that the number of cases of fatal intimate partner violence against women increased during the COVID-19 pandemic, primarily due to social distancing strategies and the implementation of lockdowns to reduce the spread of the virus. We described cases of attempted femicide and femicide in Chile before (January 2014 to February 2020) and during (March 2020 to June 2021) the pandemic. The attempted-femicide rate increased during the pandemic (incidence rate ratio: 1.22 [95% confidence interval: 1.04 to 1.43], *p* value: 0.016), while the rate of femicide cases remained unchanged. When a comparison between attempted-femicide and femicide cases was performed, being a foreigner, having an intimate partner relationship with a perpetrator aged 40 years or more, and the use of firearms during the assault were identified as factors associated independently with a higher probability of being a fatal victim in Chile. In conclusion, this study emphasizes that attempted femicide and femicide continued to occur frequently in family contexts both before and during the COVID-19 pandemic.

## 1. Introduction

Controlling and reducing cases of femicide and attempted femicide represent a challenge for authorities worldwide [1]. These fatal events have significant impacts on societies and economies and account for 64% of all female homicide cases [2]. Femicide is understood as the murder of women due to their gender and is frequently committed by current or former intimate partners (e.g., husbands, boyfriends) [3]. In 2017, the Americas reported the second-highest intimate partner/family-related homicide rate worldwide with 1.6/100,000 women, second only to Africa (3.1/100,000 women) [2].

The pandemic caused by severe acute respiratory syndrome coronarivus-2 (SARS-coV-2) coronavirus disease 2019 (COVID-19) has had negative effects on human physical/mental health, modifying people’s behavior [4,5,6,7]. The COVID-19 pandemic has been associated with an increase in intimate partner violence against women (VAW), primarily due to the implementation of lockdowns and social distancing strategies to reduce the spread of COVID-19 [8,9]. To date, studies that evaluate the direct impact of COVID-19 on the rates of femicide are scarce [10]. 

Among Latin American countries, Chile has one of the lowest rates of femicide in the region (<1 death per 100,000 women) [11]. However, cases of gender-based attempted homicide and homicide of women occur every day. This motivated the Chilean government to implement Gabriela’s law to toughen penalties for femicide and promote the elimination of VAW. Since March 2020, COVID-19 has been active in Chile, with 1,630,830 accumulated cases through August 2021, ranking sixth in Latin America. To mitigate the transmission of COVID-19 in Chile, the government designed an intervention process called “step-by-step”, which includes four stages: phase I quarantine (total lockdown), phase II transition (lockdown on weekends), phase III preparation (some activity restrictions), and phase IV initial opening (no restrictions). The stages of step-by-step intervention were implemented in each *comuna* of Chile based on the number of active cases of COVID-19 and other epidemiological criteria. In consequence, the effects of the step-by-step intervention should be evaluated with respect to aspects such as mental health, well-being, economics, and violence using a disaggregate analysis within the country.

In this study, we report the attempted femicide and femicide rates from January 2014 to June 2021 and evaluate the effects of COVID-19 on the reported cases from the second quarter of 2020. In addition, we describe the temporal (before and during COVID-19) and geographical distributions of femicide rates. Finally, due to existing social and economic inequalities in Chile, we explored through an ecological analysis the relationships between the changes in attempted femicide and femicide rates before and during COVID-19 and the implementation of the step-by-step intervention as well as to the human development index (HDI) as an indicator of socioeconomic development.

Additionally, the characteristics of the victim, perpetrator, and the event in cases of attempted femicide and femicide that occurred in Chile were assessed, comparing the cases reported before and during the COVID-19 pandemic. The risk factors associated with the transition from attempted femicide to femicide are described.

## 2. Materials and Methods

### 2.1. Study Design

This study analyzed the cases of attempted femicide and femicide from January 2014 to June 2021 reported in Chile through the National Service for Women and Gender Equality (*Servicio Nacional para la mujer y equidad de género or SERNAMEG*). According to Chilean law, killing a woman is considered femicide when the aggressor is a current or former intimate partner with whom the victim had a sexual or romantic relationship or when the cause or motivation to commit a murder is because of her gender. Consequently, it is considered attempted femicide when violence is committed against a woman with the intention of killing her and would have met the conditions to be recognized as a case of femicide if the event had been fatal [12]. A complete autopsy was performed in all femicide cases by the Legal Medical Service of Chile (*Servicio Médico Legal*, *SML*) in accordance with the guidelines of Chilean law.

Information on the number of days in each stage of the step-by step intervention was retrieved from the repository of the Chilean Minister of Science, Technology, Knowledge, and Innovation [13]. Because the stage of the step-by-step intervention was defined in each *comuna* according to the number of active cases of COVID-19 and the risk of transmission, the rows of the dataset correspond to each *comuna* and the columns to the intervention stage for each day of the study period from 28 July 2020 to 30 June 2021. 

Chile is divided into 16 regions (the first-level political division), which are subdivided into 56 provinces (the second-level political division) and these into 346 *comunas*. In our disaggregation analysis to assess whether the effect of the COVID-19 pandemic on rates of attempted femicide and femicide varied across the country, all data were summarized at a more aggregated level, and rates were calculated by province. Consequently, the average per *comuna* of days in each stage of the step-by-step intervention was used to represent each province. 

We used the HDI to investigate the effect of the province-level socioeconomic status on femicides and attempted cases. The HDI is composed of three dimensions, health, education, and economic, and is measured through the following indicators: life expectancy at birth, expected and mean years of schooling, and gross national index. The HDI is calculated with the geometric mean of normalized indices, ranging from 0 to 1 (highest development). Values of HDI below 0.55 indicate low human development; HDI values between 0.55 to 0.699, medium human development; HDI values from 0.700 to 0.799, high human development, and HDI values equal or above 0.800, very high human development. Province HDI was obtained from the last official report by the United Nations in 2017 [14], measured in 46 provinces with a range between 0.648 (Malleco Province) and 0.775 (Santiago Province).

### 2.2. Data Collection

All information on victims was gathered from the SERNAMEG database through the Chilean law of transparency and access to public information. Age, civil status, nationality, relationship with the aggressor, and number of children were analyzed for each victim. Characteristics of the event such as place, day, hour of the day, and type of weapon were also analyzed. Only the age and nationality of the aggressor were available for most events in the database. The history of previous legal complaints against the aggressor was also documented, as well as the existence of precautionary measures to stay away from the woman. Finally, how the attempted femicide or femicide event closed was characterized using the following options: arrest, escape, suicide or attempted suicide, and others.

### 2.3. Analysis

First, we calculated the rates at national and provincial levels per 100,000 women aged 15 or over. The total female population age 15 and older for each province was obtained from the website of the National Institute of Statistics (INE) based on the 2017 census [15]. Cases were subdivided into the following periods: before COVID if the event occurred between January 2014 and February 2020 and during COVID if the event was reported from March 2020 to June 2021.

Second, an INGARCH (integer-value generalized autoregressive conditional heteroscedasticity) model was used to evaluate the effects of the COVID-19 pandemic measures considering several factors (e.g., time-varying covariates, previous observations) and assuming a Poisson distribution for the numbers of femicides and attempted femicides at the national level [16]. A Poisson INGARCH (1,1) model was fitted, and the first-order autocorrelation coefficient and the first-order moving average coefficient were not significantly different from zero. For this reason, finally, the effect of COVID-19 was estimated using Poisson generalized linear models with a logit-link function using a dichotomous covariate (1: During COVID-19, 0: Before COVID-19) [17]. 

Third, differences before and during the COVID-19 pandemic in attempted femicide and femicide rates were calculated for each province. After discarding the spatial autocorrelation of the differences in rates before and during COVID-19 in Chile´s provinces with the Moran I index [18], linear mixed models were estimated using as dependent variables a transformation of rates with log(x + 1) [19].

Linear mixed models were implemented to evaluate the effects of phase I of the step-by-step intervention, HDI, and the COVID-19 pandemic on attempted femicide and femicide rates. Specifically, linear mixed models have the advantage of allowing for modeling repeated measures across time (e.g., before and during COVID-19) and assessing the effects of explanatory variables on an outcome (dependent variable) while controlling for the differences between groups (e.g., provinces). In our case, we propose a model that includes random intercepts and slopes to model differences in the reported rates of attempted femicide and femicide and differences in the effects of COVID on the outcome, taking into account the province. Formally:

$$\begin{array}{cc} y_{ij}=\beta_{0j}+\beta_{1j}COVID_{ij}+\varepsilon_{ij}, & \left(\mathrm{Level}\;1,\;\mathrm{Time}\right)\\ \beta_{0j}=\alpha_{0}+\alpha_{1}HDI_{j}+\alpha_{2}DaysPhaseI_{j}+r_{0j}, & \left(\mathrm{Level}\;2,\;\mathrm{Provinces}\right)\\ \beta_{1j}=\alpha_{3}+\alpha_{4}HDI_{j}+r_{1j}, \end{array}$$

where *i* represents the rate in a specific period (before and during COVID “Time”) and *j* a specific province. We consider one random intercept across the provinces ($\beta_{0j}$) and one random slope ($\beta_{1j}$) to estimate the effect of COVID capturing the variations across provinces. Due to the sample size, the random effect of $\beta_{1j}$ is estimated only in three groups, which were generated according to the tertiles of the differences in the rates between before and during COVID-19.

The comparison of the characteristics between the cases reported before and during the COVID-19 pandemic was performed using the Mann–Whitney nonparametric test when the variables were quantitative. Qualitative variables were compared using Pearson chi^2^ or Fisher’s exact test. Logistic regression was applied to determine the independent variables associated with femicide. The independent variable was categorized as follows: 1: femicide or 0: attempted femicide. Model selection was carried out using a backward selection methodology, including only variables with a *p* value < 0.20 in bivariate analysis. The oRs (odds ratios) were reported with 95% confidence intervals (CIs). Data were analyzed using Stata version 17.0 (StataCorp LLC, College Station, TX, USA) and R Software, version 4.1.2.

## 3. Results

**Figure 1:**
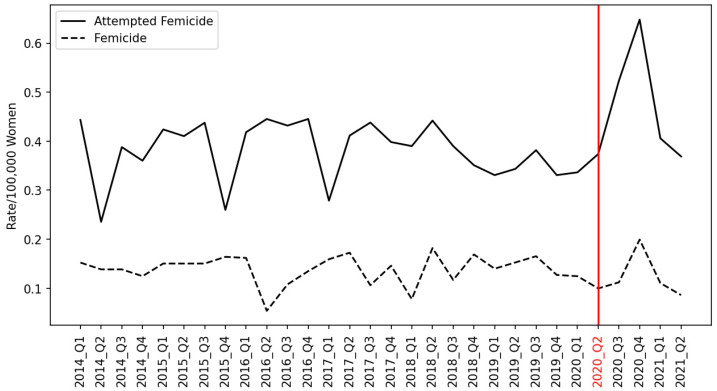
Rates of the attempted murder of women and of femicide between the first quarter of 2014 and the second quarter of 2021 in Chile.

Between January 2014 and June 2021, 1213 attempted femicide or femicide events were reported in Chile, with attempted murder and femicide rates of 12.04 and 4.27 per 100,000 women aged 15 years and over, respectively. Among these, 310 cases were homicides of women recognized as femicides, and 903 cases were attempted femicides according to Chilean law. The quarter-to-quarter rates/100,000 women of attempted femicide and femicide over the years are shown in Figure 1. The mean quarterly attempted femicide and femicide rates were 0.39 and 0.13/100,000 women, respectively. Both rates increased from 2020 Q2 to Q4, and the highest rates were reported in 2020 Q4. There was no evidence of serial correlation between attempted femicide and femicide series during the study time frame. The rate of attempted femicide increased by 22% during the COVID-19 pandemic (incidence rate ratio, IRR: 1.22 [95% CI: 1.04 to 1.43], *p* value: 0.016). The COVID-19 pandemic did not significantly affect the femicide rate in Chile (IRR: 0.87 [95% CI: 0.64 to 1.19], *p* value: 0.394). 

**Figure 2:**
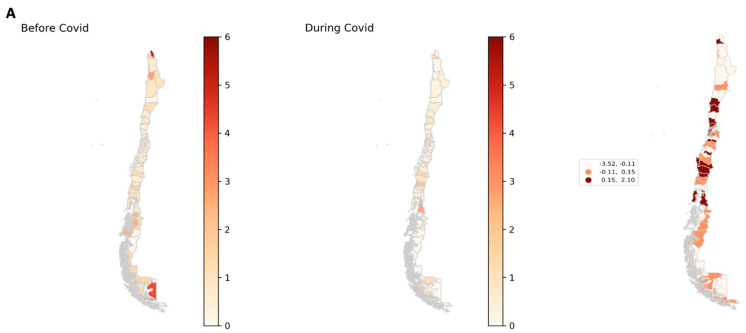

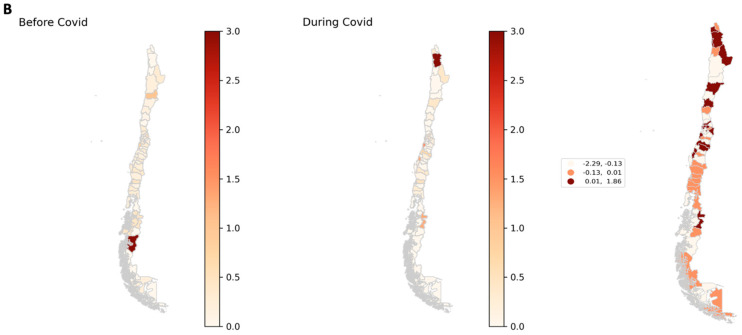
Distributions of the attempted-femicide and femicide rates before and during COVID-19 in Chile. (**A**) Mean quarterly attempted-femicide rate per 100,000 women before and during COVID-19 and tertile map of the differences in each province. (**B**) Mean quarterly femicide rate per 100,000 women before and during COVID-19 and tertile map of the differences in each province.

In Figure 2, the spatial distributions of the mean quarterly attempted-femicide and femicide rates per 100,000 women before and during the COVID-19 pandemic in each of Chile´s provinces are shown. The largest increases in mean quarterly attempted-femicide rate were reported in Palena (0.55 vs. 2.65/100,000 women), Isla de Pascua (4.05 vs. 6.02/100,000 women), and Valdivia (0.42 vs. 2.01/100,000 women), while the provinces of Parinacota (3.52 vs. 0/100,000 women), Tierra del Fuego (2.86 vs. 0/100,000 women), and Tocopilla (1.89 vs. 0/100,000 women) reported the greatest reduction in the mean quarterly rate of attempted femicide during COVID. The largest changes in mean quarterly femicide rate were reported in Capitán Prat (2.29 vs. 0/100,000 women) and Tamarugal (0 vs. 1.86/10,000 women) (Supplementary Table S1). 

The femicide model confirmed a significant interaction term between HDI and COVID (Table 1). We found that the femicide rate tended to be higher during COVID-19 with the increase in HDI. There was not a significant effect of the number of days of phase I on femicide rate. The attempted-femicide model did not reveal a significant relationship with the number of days in phase I, HDI, or COVID-19 period. 

### Characteristics of the Victims

According to the event type, differences in victim characteristics were found for age, legal marital status, relationship with the aggressor, and other variables (Table 2). There were higher proportions of women age 40 years or over (43.3%, n = 133 vs. 30.9%, n = 279), married women (42.5%, n = 130 vs. 30.3%, n = 272), women of non-Chilean nationality (10.6%, n = 33 vs. 7.4%, n = 67), and residents of rural areas (20.4%, n = 63 vs. 12.2%, n = 110) among femicide victims compared with attempted murder victims. The percentages of having children together (33.2%, n = 103 vs. 47.8%, n = 432) and previous legal complaints (32.6%, n = 101 vs. 47.3%, n = 427) were lower in femicides than in attempted murders. Aggressors of femicide more frequently committed attempted suicide and suicide (27.1%, n = 84 vs. 2.7%, n = 25). Almost half of attempted femicides and femicides were committed using knives or cutting instruments (49.2%, n = 589). However, firearms were more frequently used for femicide (17.5%, n = 52 vs. 5.9%, n = 53). In addition, the hour of day and day of the week when the events occurred were similar between attempted femicides and femicides. One third of all cases occurred from 0:00 to 6:59, and two thirds on working days. During the COVID-19 period, a total of 236 events were reported (attempted femicide: 187 cases, femicide: 49). Among these, 184 occurred after the step-by-step intervention had been implemented (33.7%, n = 62) in Chile´s *comunas* in Phase I, 27.7% (n = 51) in Phase II, 31.0% (n = 57) in Phase III, and 7.6% (n = 14) in Phase IV. The distribution of reported cases in each phase was similar between attempted femicide and femicide cases; 65.8% (n = 15) of femicides and 60.3% (n = 88) of attempted femicides took place in *comunas* during Phase I or Phase II (Table 2).

**Figure 3:**
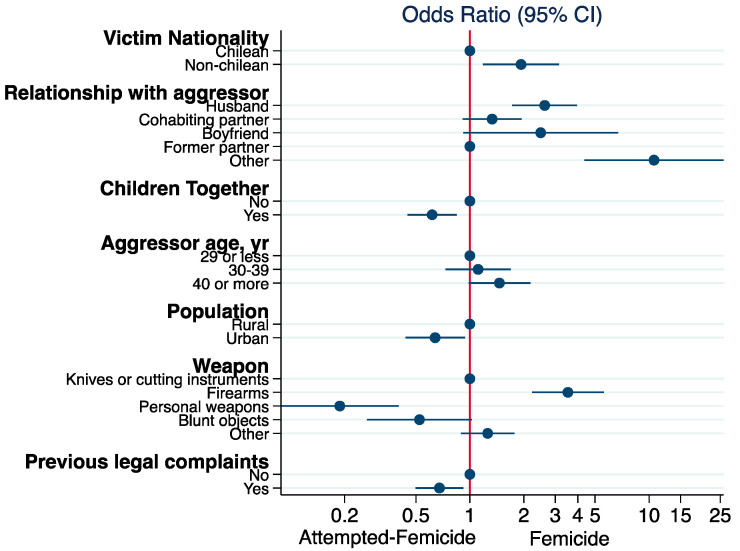
Factor risks for femicide in Chile, January 2014–June 2021.

Multivariate analysis using a logistic regression model found that femicide events were more likely to occur among foreign women (OR: 1.93, 95% CI: 1.18 to 3.15). Aggressors aged 40 or more (OR: 1.46, 95%CI: 1.00 to 2.18) and the use of firearms (OR: 3.52, 95% CI: 2.22 to 5.58) were also factors associated with femicide. Events committed by the victim’s husband (OR: 2.61, 95% CI: 1.72 to 3.97) or by a person without an intimate relationship with the victim (“Other category” OR: 10.66, 95% CI: 4.35 to 26.09) were more likely to be fatal events classified as femicides. Having children together with the aggressor (OR: 0.61, 95% CI: 0.45 to 0.84), living in urban areas (OR: 0.64, 95% CI: 0.44 to 0.94), the use of personal weapons during the assault (OR: 0.19, 95% CI: 0.09 to 0.40), and having previous legal complaints (OR: 0.68, 95% CI: 0.50 to 0.92) were associated with attempted femicide events (Figure 3).

During the COVID-19 pandemic, the proportion of femicide cases committed by people without an intimate relationship with the victim (7.7%, n = 20 vs. 20.4%, n = 10) increased. However, most aggressors were the victim´s intimate partner (e.g., husband or cohabiting partner). Among cases that occurred during COVID-19, a higher proportion of previous legal complaints (29.9%, n = 78 vs. 46.9%, n = 23) and a lower proportion of children together (36.0%, n = 94 vs. 18.4%, n = 9) were reported compared with the femicides before the pandemic. In addition, most aggressors were arrested (55.2%, n = 144 vs. 67.3%, n = 33), but a higher percentage escaped from authorities (11.1%, n = 29 vs. 22.4%, n = 11). In contrast, the proportion of femicide perpetrators who committed suicide diminished during COVID-19 (28.3%, n = 74 vs. 10.2%, n = 10.2) (Supplementary Table S2). 

Regarding the characterization of attempted femicide cases before and after the COVID-19 pandemic, the percentage of victims with single marital status (59.9%, n = 428 vs. 69.0%, n = 127), femicides committed by cohabiting partner (45.1%, n = 323 and 51.3%, n = 96), the use of personal weapons (12.6%, n = 90 vs. 27.0%, n = 50), and events occurring from 20:00 to 23:59 (18.8%, n = 134 vs. 27.8%, n = 52) significantly increased during COVID-19, as well as attempts by aggressors with previous legal complaints (44.3%, n = 317 vs. 58.8%, n = 110). No statistical differences in victim or aggressor age, victim nationality, assault location, type of population, or day of the week were found between femicide attempts and cases perpetrated before and during the COVID-19 pandemic (Supplementary Table S1). 

## 4. Discussion

VAW is recognized as a violation of human rights and can take many forms, including verbal, physical, and sexual abuse and even murder. Femicide is the most extreme type of VAW that occurs solely because the victims are women and is related primarily to social and economic inequalities [1]. Since the beginning of the COVID-19 pandemic, experts and global organizations have hypothesized that a significant increase in the number of VAW cases, including femicides, was likely to be observed due to intensified tensions in the domestic context and the restriction of movement [9,10]. In this study, the analysis revealed that the highest rates of attempted femicide and femicide in Chile were observed at 2020 Q4, corresponding to nine months after the first report of COVID-19 in Chile. Furthermore, we found a significant change in the quarterly attempted femicide rate measured at the country level from Q2 2020 to Q2 2021, the COVID-19 period, with a 22% increase compared with the difference from Q1 2014 to Q1 2020. The femicide rate at country level remained constant before and after COVID-19.

Historically and as shown in our results, most femicides and attempted femicides are committed by people close to the victim, and approximately half of the events take place at the victim’s home. In light of this information, and due to the implementation of stay-at-home measures since the beginning of the COVID-19 pandemic, alerts have been generated from international organizations and politicians to prevent the intensification of VAW. However, from the limited available data published during the current pandemic, three facts have been presented: (1) an increase in the number of calls from women who reported being victims of intimate partner violence [20], (2) an overall increase in domestic or intimate partner VAW, as well as the severity of injuries (e.g., attempted femicide) [8,21,22,23], and (3) a reduction in fatal victims of VAW or femicide, as seen in this study [8]. 

Despite growing concern about femicide behavior worldwide, research based on the analysis of public or official data is still limited. In a Latin American context, reports from Peru have described homicide rates among women during the COVID-19 period, not specifically femicides, as showing a decrease in the number of cases during the early weeks of the pandemic, returning to rates equivalent to those reported in previous periods [24]. This U-shaped trend was also reported in Mexico regarding all crimes against women including sexual crimes, domestic violence, and murder. However, similar to this study, a constant femicide rate was described in Mexico during and after the intervention measures for the COVID-19 pandemic [25]. Evidence from Spain after monitoring several indicators associated with VAW from January 2015 to September 2020 revealed that the number of women killed by their intimate partners decreased during the COVID-19-induced lockdown, which coincided with an increase in the number of telephone calls for help for VAW [8].

Another study from Turkey evaluating the effects of COVID-19 distance measures on female homicides between 2014 and 2019 reported a total reduction (57%) in murders of women committed by their intimate partners due to COVID-19 interventions. Moreover, the highest reduction (83%) was described during the days of curfew implementation [26]. Similarly, a metaregression analysis that involved information from 23 countries in the Americas, Europe, the Middle East, and Asia described that the overall homicide rate without discrimination by sex diminished during the spread of COVID-19 due to restriction-of-movement interventions, with a gradual return to previous rates [27]. All these findings have opened the door to a new line of research to evaluate if the distancing measures could have had a beneficial effect on homicides of women over time.

Interestingly, when the disaggregate analyses were carried out using the mean quarterly rates reported by Chile´s provinces, an inverse effect of COVID-19 was observed. On the one hand, using a mixed-model approach, we did not find a significant impact of COVID-19 or HDI in the attempted femicide rates. However, on the other hand, the significant positive coefficient of the interaction between the COVID-19 pandemic and HDI on Chile´s province-level femicide rate means that there was a rise during the pandemic in provinces with better socioeconomic conditions. This finding is similar to that reported in Brazil [28], and it could be correlated with the results provided by Gozzi et al. [29], who described that people living in places with a higher HDI in Chile tend to be more adherent to human mobility restrictions, consistent with the current hypothesis of the COVID-19 impact on femicides. Nonetheless, the results from this study did not support the existence of a direct effect of COVID-19 confinement measures, represented by the average number of days in phase I during the restriction intervention implemented in Chile, on the numbers of cases of attempted femicide and femicide. 

In the Chilean context, concern about the prevention of cases of VAW and femicides has increased during the last decade. Gabriela´s law was implemented in early March 2020 at the same time that the first case of COVID-19 in Chile was identified. Consequently, the impacts of COVID-19 and of Gabriela’s law on attempted femicides and femicides could have been confounded. In addition, a recent report from the Millennium Institute for the Study of Life Course and Vulnerability found a 149% increase in calls reporting VAW in 2020 in comparison with 2019, reaching a peak during the early COVID-19 lockdowns between April and May 2020. However, the number of official complaints decreased [30]. These facts could be explained because most of the women were forced to live longer hours in the same house with their aggressor, and this, combined with reduced mobility, could have restricted women’s easy access to the police and authorities.

We addressed two questions here: first, whether differences exist between cases of attempted femicide and femicide and second, whether the typical behavior of these fatal events changed as a consequence of the COVID-19 pandemic in terms of sociodemographic and aggression characteristics. Being a foreigner, having an intimate partner relationship with a perpetrator aged 40 years or more, and the use of firearms during the assault were identified as independent factors associated with a higher probability of being a fatal victim in Chile. Although the victim’s age was not found to be a determining factor related to femicides, approximately two thirds of attempted femicide and femicide victims were under 40 years of age, which is similar to the ages reported in other international series [31,32]. Furthermore, as reported in Italy [31,33], a high proportion of women aged 40 years or older was found among the fatal cases that occurred in Chile, which explains the finding related to the aggressor’s age.

Data showed that suicide among people who committed femicides in Chile occurred in up to 25% of the events during the study period, which is denominated in the literature as “femicide–suicide” [34]. Contrary to beliefs, this proportion fell to 10.2% during the COVID period, and this observed effect on femicide–suicide in Chile is consistent with some studies in which a non-increase in the suicide rate was described [35]. This femicide–suicide phenomenon generally appears to be explained by a depression diagnosis or prior suicide threats before the woman is killed by her intimate partner [36].

Relevant findings during the COVID-19 pandemic were the non-increase in the proportion of crimes that occurred in either the victim’s or the aggressor’s home and the increases in the numbers of crimes committed by people with antecedents of legal complaints and of attempted femicide cases in which women were kicked, slapped, or hit. These two last findings suggest that the current pandemic intensified violent behaviors and could have played a role in the development of both fatal crimes [8,22].

As our results show, in Chile, a foreign woman is at a higher risk of femicide. This fact has also been described in other countries; for example, in Italy, the risk of visiting the emergency room for intentional injuries is three times higher among foreign women [37]. In Spain, foreign women have a higher risk of death due to intimate partner violence, with an estimate between 2 to 8 times higher than that among Spanish women [38]. Although all women who live in a specific place must have the guarantee that their human rights will be protected by the authorities and the laws, we consider that foreign women represent a vulnerable group and are more likely to suffer violence. Therefore, it is necessary to generate calls to action in order to close the gaps in safety between foreign and non-foreign women in Chile and around the world.

In a similar study conducted in Peru that described the attempted femicide and femicide cases during pre-quarantine, quarantine, and restrictive-measures periods during the COVID-19 pandemic in 2020, there was a significantly higher proportion of fatal cases during the quarantine period (32.6%) compared with the other periods (pre-quarantine: 19.9% and restrictive measures: 22.0%). Similar to our findings, the attempted-femicide risk in Peru increased with antecedents of physical and psychological violence (i.e., previous legal complaints), as well as living in urban areas during the three scenarios [39].

In Chile, the definition of femicide has evolved through two local laws, enacted in 2010 (the antifemicide law) and 2020 (Gabriela´s law). These laws have made it possible to broaden the term to include any intentional homicide of a woman perpetrated in situations where the victim has less power or is in a vulnerable position in relation to the aggressor for being a woman without considering the existing relationship between the women and perpetrator. Initially, violence against women committed by their current or former intimal partners was considered femicide, but this excluded other cases involving criminal sexual acts perpetrated by non-intimate partners. Currently, it is difficult to establish specific conditions for classifying the murder of a woman as femicide in Chile, leading to the legal application of its definition depending on how judges and courts interpret the law, specifically when the aggressor does not have a direct relationship with the victim.

This study has limitations. First, although we performed an integrative analysis of the behavior of attempted femicide and femicide rates, the relationships we found at the aggregate (e.g., country) level may not explain the true effects at an individual level, making it difficult to extrapolate our results. Second, due to the differences in the designs of the step-by-step lockdowns as implemented in Chile from *comuna* to *comuna*, it may be complex to evaluate the real impacts of this intervention on femicides. Moreover, when we explored the data for each *comuna*, many places reported zero or few femicides or attempted femicides, which could have changed the relationships with the other ecological variables in the statistical models; therefore, all disaggregate analyses were performed using the second-level division in Chile, provinces. Second, the sample included only cases reported in Chile, so our results may not be generalizable to all countries. However, this study represents a starting point for understanding the characteristics of these fatal events during the COVID-19 pandemic. Third, because all data used in this study were obtained from official reports, some interesting variables associated with VAW in the literature were not analyzed, for example, socioeconomic status or occupation.

## 5. Conclusions

In conclusion, our findings suggest that the effects of COVID-19 on attempted femicide and femicide rates in Chile need to be evaluated according to the economic, cultural, and social aspects of each specific province. In Chile, the attempted femicide rate increased from the baseline rate reported before the COVID-19 pandemic. Although the rate of femicide in Chile was constant during the observation period, a particular effect was observed that suggested a negative impact of COVID-19 on the fatality rate in places with high socioeconomic levels. The present study has emphasized that attempted femicide and femicide continue to occur frequently in family contexts and were committed in intimate relationships both before and during the COVID-19 pandemic. We believe that our results represent a starting point for improving the understanding of femicides in Chile and for moving toward the creation and implementation of interventions that will reduce and control all types of VAW.

## Figures and Tables

**Table 1:** Mixed models to evaluate the relationships of COVID-19 and HDI with the attempted-femicide and femicides rates.

Variable	Attempted Femicide			Femicide		
	Regression Coefficients Estimated (Fixed Effects)	95%CI	*p*-Value	Regression Coefficients Estimated (Fixed Effects)	95%CI	*p*-Value
Phase I Days	0.00003	−0.0014 to 0.0015	0.961	−0.0004	−0.001 to 0.0002	0.198
HDI	−0.543	−2.165 to 1.078	0.503	−0.636	−1.513 to 0.239	0.150
COVID	−0.007	−0.094 to 0.080	0.871	−1.288	−2.413 to −0.163	0.026
Interaction COVID × HDI	-	-	-	1.762	0.179 to 3.345	0.029

Coefficients were estimated in linear mixed models using as dependent variables a transformation (log (x + 1)) of the attempted-femicide and femicide rates. CI: Confidence interval. HDI: human development index (HDI).

**Table 2:** Characteristics of the event, victim, and aggressor between the attempted femicide and femicide cases.

Characteristics	Total (n = 1213)	Femicide (n = 310)	Attempted Femicide (n = 903)	*p*-Value
Victim age, years				
No.	1210	307	903	
Median (IQR)	34 (27 to 44)	37 (28 to 48)	33 (27 to 42)	
15–29 years	411 (34.0)	95 (30.9)	316 (35.0)	0.000 ^b^
30–39 years	387 (32.0)	79 (25.7)	308 (34.1)	
40+ years	412 (34.0)	133 (43.3)	279 (30.9)	
Victim legal marital status, n (%)				
No.	1205	306	899	
Married	402 (33.4)	130 (42.5)	272 (30.3)	
Divorced	80 (6.6)	23 (7.5)	57 (6.3)	
Separated	1 (0.1)	0 (0.0)	1 (0.1)	0.000 ^c^
Single	701 (58.2)	146 (47.7)	555 (61.7)	
Widowed	21 (1.7)	7 (2.3)	14 (1.6)	
Victim Nationality, (%)				
Chilean	1113 (91.8)	277 (89.3)	836 (92.6)	0.075 ^d^
Non-Chilean	100 (8.2)	33 (10.6)	67 (7.4)	
Relationship with aggressor, n (%)				
Husband	269 (22.2)	94 (30.3)	175 (19.4)	
Cohabiting partner	536 (44.2)	117 (37.7)	419 (46.4)	
Boyfriend	23 (1.9)	9 (2.9)	14 (1.5)	
Former partner	345 (28.4)	60 (19.3)	285 (31.6)	0.000 ^c^
Other	40 (3.3)	30 (6.7)	10 (1.1)	
Children Together, n (%)	535 (44.1)	103 (33.2)	432 (47.8)	0.000 ^d^
Aggressor age, years				
Median (IQR)	37 (29 to 48)	40 (32 to 54)	36 (29 to 46)	0.000 ^b^
Aggressor Nationality, n (%)				
Chilean	1121 (92.4)	279 (90.0)	842 (93.2)	0.063 ^d^
Non-Chilean	92 (7.6)	31 (10.0)	61 (6.8)	
Result, n (%)				
Arrest	851 (70.2)	177 (57.1)	674 (74.6)	
Escape	237 (19.5)	40 (12.9)	197 (21.8)	
Suicide	101 (8.3)	79 (25.5)	22 (2.4)	0.000 ^c^
Attempted Suicide	8 (0.7)	5 (1.6)	3 (0.3)	
Other	16 (1.3)	9 (2.9)	7 (0.8)	
Place, n (%)				
No.	1211	310	901	
Home	646 (53.3)	165 (53.2)	481 (53.4)	
Victim Residence	231 (19.1)	51 (16.4)	180 (20.0)	
Aggressor Residence	37 (3.1)	10 (3.2)	27 (3.0)	0.035 ^d^
Other Residence	68 (5.6)	28 (9.0)	40 (4.4)	
Street	229 (18.9)	56 (18.1)	173 (19.2)	
Population, n (%)				
No.	1211	309	902	
Rural	173 (14.3)	63 (20.4)	110 (12.2)	0.000 ^d^
Urban	1038 (85.7)	246 (79.6)	792 (87.8)	
Day of week, n (%)				
Working days	765 (63.1)	204 (65.8)	561 (62.1)	0.247 ^d^
Non-working days	448 (36.9)	106 (34.2)	342 (37.9)	
Hour of day, n (%)				
0:00 to 6:59	400 (33.0)	92 (29.7)	308 (34.1)	
7:00 to 12:59	268 (22.1)	75 (24.2)	193 (21.4)	
13:00 to 19:59	301 (24.8)	85 (27.4)	216 (23.9)	0.289 ^d^
20:00 to 23:59	244 (20.1)	58 (18.7)	186 (20.6)	
Weapon, n (%)				
No.	1197	298	899	
Knives or cutting instruments	589 (49.2)	145 (48.7)	444 (49.4)	
Firearms	105 (8.8)	52 (17.5)	53 (5.9)	
Personal weapons (hands, feet)	149 (12.4)	9 (3.0)	140 (15.6)	0.000 ^d^
Blunt objects	79 (6.6)	12 (4.0)	67 (7.4)	
Other or not reported	275 (23.0)	80 (26.8)	195 (21.7)	
Previous legal complaints, n (%)				
Yes	528 (43.5)	101 (32.6)	427 (47.3)	0.000 ^d^
Yes (Aggressor)	270 (22.3)	49 (15.8)	221 (24.5)	0.002 ^d^
Precautionary measure (Aggressor)	136 (11.2)	33 (10.7)	103 (11.4)	0.727 ^d^
Period, n (%)				
Before COVID-19	977 (80.5)	261 (84.2)	716 (79.3)	-
During COVID-19	236 (19.5)	49 (15.8)	236 (20.7)	
Cases in Step-by-Step ^a^, n (%)				
No.	184	38	146	
Phase I	62 (33.7)	10 (26.3)	52 (35.6)	
Phase II	51 (27.7)	15 (39.5)	36 (24.7)	-
Phase III	57 (31.0)	12 (31.6)	45 (30.8)	
Phase IV	14 (7.6)	1 (2.6)	13 (8.9)	

IQR: Interquartile range; yr: years; No.: Number of observations with non-missing data. ^a^ Information available from 28 July 2020 to 30 June 2021. *p*-Values indicating differences between groups obtained using ^b^ Kruskal–Wallis, ^c^ Fisher´s exact, and ^d^ chi-square tests.

## Data Availability

The dataset is available upon reasonable request from the corresponding author.

## Supplementary Materials

The following supporting information can be downloaded at: https://www.mdpi.com/article/10.3390/ijerph19138012/s1, Table S1. Mean quarterly rates/100,000 women before and during the COVID-19 pandemic; Table S2. Characteristics of the event, victim and aggressor between cases that occurred before and during the COVID-19 pandemic.

## References

[1] World Health Organization (2021). Violence against Women Prevalence Estimates, 2018: Global, Regional and National Prevalence Estimates for Intimate Partner Violence against Women and Global and Regional Prevalence Estimates for Non-Partner Sexual Violence Against Women.

[2] UNODC (2019). The Global Study on Homicide 2019.

[3] World Health Organization (2012). Understanding and Addressing Violence against Women.

[4] Hampshire A., Hellyer P.J., Soreq E., Mehta M.A., Loannidis K., Trender W., Grant J.E., Chamberlain S.R. (2021). Associations between dimensions of behaviour, personality traits, and mental-health during the COVID-19 pandemic in the United Kingdom. Nat. Commun..

[5] Rose A., Walmsley T., Wei D. (2021). Spatial transmission of the economic impacts of COVID-19 through international trade. Lett. Spat. Resour. Sci..

[6] Manica M., Guzzetta G., Riccardo F., Valenti A., Poletti P., Marziano V., Trentini F., Andrianou X., Mateo-Urdiales A., del Manso M. (2021). Impact of tiered restrictions on human activities and the epidemiology of the second wave of COVID-19 in Italy. Nat. Commun..

[7] Tanaka T., Okamoto S. (2021). Increase in suicide following an initial decline during the COVID-19 pandemic in Japan. Nat. Hum. Behav..

[8] Vives-Cases C., Parra-Casado D.L., Estévez J.F., Torrubiano-Domínguez J., Sanz-Barbero B. (2021). Intimate Partner Violence against Women during the COVID-19 Lockdown in Spain. Int. J. Environ. Res. Public Health.

[9] Sánchez O.R., Vale D.B., Rodrigues L., Surita F.G. (2020). Violence against women during the COVID-19 pandemic: An integrative review. Int. J. Gynecol. Obstet..

[10] Weil S. (2020). Two Global Pandemics: Femicide and COVID-19. Trauma Mem..

[11] Cepal N.U. (2020). Addressing Violence against Women and Girls during and after the COVID-19 Pandemic Requires FINANCING, RESPONSES, PREVENTION AND DATA COMPILATION.

[12] Sernameg Femicidios. https://www.sernameg.gob.cl/?page_id=27084.

[13] Ministerio de Ciencia Tecnología Conocimiento e Innovación Datos COVID-19. https://github.com/MinCiencia/Datos-COVID19/tree/master/output/producto74.

[14] PNUD (2018). Desigualdad Regional en CHILE. Ingresos, Salud y Educación en Perspectiva Territorial.

[15] INE Censo de Población y Vivienda. https://www.ine.cl/estadisticas/sociales/censos-de-poblacion-y-vivienda/poblacion-y-vivienda.

[16] Ferland R., Latour A., Oraichi D. (2006). Integer-Valued GARCH Process. J. Time Ser. Anal..

[17] Agresti A. (2018). An Introduction to Categorical Data Analysis.

[18] Cliff A.D. (1973). Spatial Autocorrelation.

[19] Cnaan A., Laird N.M., Slasor P. (1997). Using the general linear mixed model to analyse unbalanced repeated measures and longitudinal data. Stat. Med..

[20] Viero A., Barbara G., Montisci M., Kustermann K., Cattaneo C. (2020). Violence against women in the Covid-19 pandemic: A review of the literature and a call for shared strategies to tackle health and social emergencies. Forensic Sci. Int..

[21] Rhodes H.X., Petersen K., Lunsford L., Biswas S. (2020). COVID-19 Resilience for Survival: Occurrence of Domestic Violence During Lockdown at a Rural American College of Surgeons Verified Level One Trauma Center. Cureus.

[22] Nittari G., Sagaro G., Feola A., Scipioni M., Ricci G., Sirignano A. (2021). First Surveillance of Violence against Women during COVID-19 Lockdown: Experience from “Niguarda” Hospital in Milan, Italy. Int. J. Environ. Res. Public Health.

[23] Gosangi B., Park H., Thomas R., Gujrathi R., Bay C.P., Raja A.S., Seltzer S.E., Balcom M.C., McDonald M.L., Orgill D.P. (2021). Exacerbation of Physical Intimate Partner Violence during COVID-19 Pandemic. Radiology.

[24] Calderon-Anyosa R.J., Bilal U., Kaufman J.S. (2021). Variation in Non-external and External Causes of Death in Peru in Relation to the COVID-19 Lockdown. Yale J. Biol. Med..

[25] Hoehn-Velasco L., Silverio-Murillo A., de la Miyar J.R.B. (2021). The great crime recovery: Crimes against women during, and after, the COVID-19 lockdown in Mexico. Econ. Hum. Biol..

[26] Asik G.A., Ozen E.N. (2021). It takes a curfew: The effect of Covid-19 on female homicides. Econ. Lett..

[27] Nivette A.E., Zahnow R., Aguilar R., Ahven A., Amram S., Ariel B., Burbano M.J.A., Astolfi R., Baier D., Bark H.-M. (2021). A global analysis of the impact of COVID-19 stay-at-home restrictions on crime. Nat. Hum. Behav..

[28] de Sá Y.R.C., Moi P.C.P., Galvão N.D., da Silva A.M.C., Moi G.P. (2021). The geography of femicide in Sergipe, Brazil: Matriarchy, human development, and income distribution. Rev. Bras. Epidemiol..

[29] Gozzi N., Tizzoni M., Chinazzi M., Ferres L., Vespignani A., Perra N. (2021). Estimating the effect of social inequalities on the mitigation of COVID-19 across communities in Santiago de Chile. Nat. Commun..

[30] MIPP Violencia Contra la Mujer en la Cuarentena: Denuncias Bajaron 9.6% y Llamadas de Auxilio Aumentaron 43.8%. https://www.mipp.cl/miradas/2021/03/11/violencia-contra-la-mujer-en-la-cuarentena-denuncias-bajaron-96-y-llamadas-de-auxilio-aumentaron-438/.

[31] Sorrentino A., Guida C., Cinquegrana V., Baldry A. (2020). Femicide Fatal Risk Factors: A Last Decade Comparison between Italian Victims of Femicide by Age Groups. Int. J. Environ. Res. Public Health.

[32] Cullen P., Vaughan G., Li Z., Price J., Yu D., Sullivan E. (2018). Counting Dead Women in Australia: An In-Depth Case Review of Femicide. J. Fam. Violence.

[33] Biehler-Gomez L., Maggioni L., Tambuzzi S., Kustermann A., Cattaneo C. (2022). Twenty years of femicide in Milan: A retrospective medicolegal analysis. Sci. Justice.

[34] Dayan H. (2018). Sociocultural Aspects of Femicide-Suicide: The Case of Israel. J. Interpers. Violence.

[35] Tandon R. (2021). COVID-19 and suicide: Just the facts. Key learnings and guidance for action. Asian J. Psychiatry.

[36] Koziol-McLain J., Webster D., McFarlane J., .Block C.R., Ulrich Y., Glass N., Campbell J.C. (2006). Risk factors for femicide-suicide in abusive relationships: Results from a multisite case control study. Violence Vict..

[37] Farchi S., Polo A., Asole S., Ruggieri M.P., Di Lallo D. (2013). Use of emergency department services by women victims of violence in Lazio region, Italy. BMC Women’s Health.

[38] Vives-Cases C., Álvarez-Dardet C., Torrubiano-Domínguez J., Gil-González D. (2008). Mortalidad por violencia del compañero íntimo en mujeres extranjeras residentes en España (1999–2006). Gac. Sanit..

[39] Bardales Mendoza O.T., Meza Díaz R., Carbajal M. (2022). Feminicide Violence Before and During the COVID-19 Health Emergency. Violence Gend..

