# Supplementary material for: Femicide and Attempted Femicide before and during the COVID-19 Pandemic in Chile
Source: Int J Environ Res Public Health. 2022 Jun 30;19(13):8012. doi: 10.3390/ijerph19138012 (PMC9265640; doi:10.3390/ijerph19138012)
Supplement: Supplementary file 1 [file ijerph-19-08012-s001.zip › ijerph-1794710-supplementary.pdf]

Table S1. Mean quarterly rates/100,000 women before and during the COVID-19 pandemic

Region	Province	Before Femicide	COVID Femicide	Before Attempted	COVID Attempted
**Antofagasta**	Antofagasta	0.15	0.00	0.58	0.33
	El Loa	0.23	0.25	0.70	0.51
	Tocopilla	0.00	0.00	1.89	0.00
**Arica y Parinacota**	Arica	0.09	0.20	0.96	1.41
	Parinacota	0.00	0.00	3.52	0.00
**Atacama**	Copiapó	0.11	0.25	0.80	0.50
	Chañaral	0.78	0.00	0.00	0.00
	Huasco	0.13	0.00	0.39	0.63
**Aysén**	Coyhaique	0.34	0.81	0.86	0.81
	Aysén	0.34	0.00	1.70	0.00
	Capitán Prat	2.29	0.00	0.00	0.00
	General Carrera	0.00	0.00	0.00	0.00
**Biobío**	Concepción	0.11	0.13	0.24	0.18
	Arauco	0.23	0.00	0.17	0.56
	Bíobío	0.17	0.00	0.45	0.46
**Coquimbo**	Elqui	0.06	0.09	0.59	0.87
	Choapa	0.22	0.00	0.33	0.52
	Limarí	0.00	0.00	0.39	0.26
**La Araucanía**	Cautín	0.14	0.06	0.29	0.96
	Malleco	0.28	0.22	0.74	1.35
**Los Lagos**	Llanquihue	0.21	0.11	0.66	0.22
	Chiloé	0.17	0.00	0.62	0.81
	Osorno	0.24	0.19	0.68	0.78
	Palena	0.00	0.00	0.55	2.65
**Los Ríos**	Valdivia	0.36	0.31	0.42	2.01
	Ranco	0.10	0.00	1.01	0.99
**Magallanes**	Magallanes	0.15	0.00	0.95	1.03
	Antártica Chilena	0.00	0.00	0.00	0.00

	Tierra del Fuego	0.00	0.00	2.87	0.00
	Última Esperanza	0.00	0.00	0.86	0.00
**Maule**	Talca	0.11	0.43	0.21	0.21
	Cauquenes	0.16	0.00	0.80	0.00
	Curicó	0.20	0.00	0.39	0.31
	Linares	0.13	0.16	0.40	0.16
**Metropolitana**	Santiago	0.11	0.08	0.22	0.28
	Cordillera	0.11	0.14	0.21	0.21
	Chacabuco	0.15	0.32	0.35	0.00
	Maipo	0.23	0.00	0.33	0.63
	Melipilla	0.25	0.00	0.20	0.23
	Talagante	0.13	0.15	0.26	0.44
**Ñuble**	Diguillín	0.11	0.00	0.26	0.14
	Itata	0.17	0.83	0.34	0.00
	Punilla	0.26	0.00	0.79	1.26
**O´Higgins**	Cachapoal	0.24	0.07	0.67	0.28
	Cardenal Caro	0.63	0.99	0.42	0.00
	Colchagua	0.09	0.00	0.35	0.62
**Tarapaca**	Iquique	0.07	0.15	1.14	1.16
	Tamarugal	0.00	1.86	0.43	0.00
**Valparaíso**	Valparaíso	0.02	0.40	0.35	0.75
	Isla de Pascua	0.00	0.00	4.05	6.02
	Los Andes	0.45	0.00	0.45	0.00
	Petorca	0.24	0.00	0.00	0.58
	Quillota	0.05	0.22	0.37	0.44
	San Antonio	0.34	0.00	0.62	0.79
	San Felipe	0.19	0.29	0.56	0.58
	Marga Marga	0.08	0.12	0.16	0.37

Table S2. Characteristics of the event, victim and aggressor between cases that occurred before and during the COVID-19 pandemic

	**Femicide**			**Attempted**		
**Characteristics**	**Before (n=261)**	**During (n=49)**	**P value**	**Before (n=716)**	**During (n=187)**	**P value**
**Victim age, yr**						
**No.**	258	49		716	187	
**Median±IQR**	37.5 (28 to 49)	33 (22 to 46)	0.124	33 (27 to 43)	33 (26 to 41)	0.193
**15-29 yr**	74 (28.7)	21 (42.9)		247 (34.5)	69 (36.9)	
**30-39 yr**	70 (27.1)	9 (18.4)		246 (34.4)	62 (33.2)	
**40+ yr**	114 (44.2)	19 (38.8)		223 (31.1)	56 (29.9)	
**Victim legal marital status, n (%)**						
**No.**	258	48		715	184	
**Married**	115 (44.6)	15 (31.2)		235 (32.9)	37 (20.1)	
**Divorced**	22 (8.5)	1 (2.1)	0.068	40 (5.6)	17 (9.2)	0.001
**Separated**	0 (0.0)	0 (0.0)		0 (0.0)	1 (0.5)	
**Single**	116 (44.6)	31 (64.6)		428 (59.9)	127 (69.0)	
**Widowed**	6 (2.3)	1 (2.1)		12 (1.7)	2 (1.1)	
**Victim Nationality, (%)**						
**Chilean**	235 (90.0)	42 (85.7)	0.368	665 (92.9)	171 (91.4)	0.505
**Non-Chilean**	26 (10.0)	7 (14.3)		51 (7.19	16 (8.6)	
**Relationship with aggressor, n (%)**						
**Husband**	86 (32.9)	8 (16.3)		151 (21.1)	24 (12.8)	
**Cohabiting partner**	97 (37.2)	20 (40.8)	0.023	323 (45.1)	96 (51.3)	0.020
**Boyfriend**	7 (2.7)	2 (4.1)		9 (1.3)	5 (2.7)	
**Former partner**	51 (19.5)	9 (18.4)		227 (31.7)	58 (31.0)	
**Other**	20 (7.7)	10 (20.4)		6 (0.8)	4 (2.1)	
**Children Together, n (%)**	94 (36.0)	9 (18.4)	0.016	357 (49.9)	75 (40.1)	0.017
**Aggressor age, yr**						
**Median (IQR)**	40 (32 to 53)	35 (28 to 54)	0.173	37 (29 to 46)	35 (27 to 45)	0.051
**Aggressor Nationality, n (%)**						
**Chilean**	239 (91.6)	40 (81.6)	0.064	671 (93.7)	171 (91.4)	0.270
**Non-Chilean**	22 (8.4)	9 (18.4)		45 (6.3)	16 (8.6)	
**Result, n (%)**						
**Arrest**	144 (55.2)	33 (67.3)		531 (74.2)	143 (76.5)	
**Escape**	29 (11.1)	11 (22.4)	0.011	158 (22.1)	39 (20.9)	0.854
**Suicide**	74 (28.3)	5 (10.2)		19 (2.6)	3 (1.6)	
**Attempted Suicide**	5 (1.9)	0 (0.0)		2 (0.3)	1 (0.5)	
**Other**	9 (3.4)	0 (0.0)		6 (0.8)	1 (0.5)	
**Place, n (%)**						
**No.**	261	49		716	185	
**Home**	143 (54.8)	22 (4.9)		382 (53.3)	99 (53.5)	
**Victim Residence**	40 (15.3)	11 (22.4)	0.540	138 (19.3)	42 (22.7)	0.249
**Aggresor Residence**	9 (3.4)	1 (2.0)		19 (2.6)	8 (4.3)	
**Other Residence**	22 (8.4)	5 (12.2)		36 (5.0)	4 (2.2)	
**Street**	47 (18.0)	9 (18.4)		141 (19.7)	32 (17.3)	
**Population, n (%)**						

Supplementary information
Femicide and attempted-femicide before and during the COVID-19 pandemic in Chile

**No.**<br>**Rural**<br>**Urban**	261<br>54 (20.7)<br>207 (79.3)	48<br>9 (18.8)<br>39 (81.2)	0.759	716<br>83 (11.6)<br>633 (88.4)	186<br>27 (14.5)<br>159 (85.5)	0.278
**Day of week, n (%)**<br>**Working days**<br>**Non-working days**	 85 (32.6)<br>176 (67.4)	 21 (42.9)<br>28 (57.1)	0.164	 268 (37.4)<br>448 (62.6)	 74 (39.6)<br>113 (60.4)	0.591
**Hour of day, n (%)**<br>**0:00 to 6:59**<br>**7:00 to 12:59**<br>**13:00 to 19:59**<br>**20:00 to 23:59**	 81 (31.0)<br>60 (23.0)<br>71 (27.2)<br>49 (18.8)	 11 (22.4)<br>15 (30.6)<br>14 (28.6)<br>9 (18.4)	0.563	 245 (34.2)<br>160 (22.3)<br>177 (24.7)<br>134 (18.7)	 63 (33.7)<br>33 (17.6)<br>39 (20.9)<br>52 (27.8)	0.038
**Weapon, n (%)**<br>**No.**<br>**Knives or cutting instruments**<br>**Firearms**<br>**Personal weapons (hands, feet)**<br>**Blunt objects**<br>**Other or not reported**	 255<br>126 (49.4)<br>45 (17.6)<br>7 (2.7)<br>9 (3.5)<br>68 (26.7)	 43<br>19 (44.2)<br>7 (16.3)<br>2 (4.6)<br>3 (7.0)<br>12 (27.9)	0.659	 714<br>359 (50.3)<br>38 (5.3)<br>90 (12.6)<br>57 (8.0)<br>170 (23.8)	 185<br>85 (45.9)<br>15 (8.1)<br>50 (27.0)<br>10 (5.4)<br>25 (13.5)	0.000
**Previous legal complaints, n (%)**<br>**Yes**<br>**Yes (Aggressor)**<br>**Precautionary measure (Aggressor)**	 78 (29.9)<br>32 (12.3)<br>28 (10.7)	 23 (46.9)<br>17 (34.7)<br>5 (10.4)	 0.019<br>0.000<br>0.949	 317 (44.3)<br>157 (21.9)<br>88 (12.3)	 110 (58.8)<br>64 (34.2)<br>15 (8.0)	 0.000<br>0.000<br>0.102

IQR: Interquartile range
